# Interferon-β-induced pulmonary sarcoidosis in a 30-year-old woman treated for multiple sclerosis: a case report

**DOI:** 10.1186/1752-1947-6-344

**Published:** 2012-10-08

**Authors:** Nayia Petousi, Enson C Thomas

**Affiliations:** 1 Chest Clinic, Ombersley House, Bedford Hospital, Kempston Road, Bedford, MK42 9DJ, UK; 2 Department of Physiology, Anatomy and Genetics, University of Oxford, Parks Road, Oxford, OX1 3PT, UK

## Abstract

**Introduction:**

With the increasing use of recombinant α and β interferon therapy for the treatment of various disorders, cases of interferon-associated sarcoidosis have been reported in the literature. The majority of these have been cases of interferon-α-induced sarcoidosis. We present the first case, to the best of our knowledge, of interferon-induced pulmonary sarcoidosis in a patient whose multiple sclerosis was treated with interferon-β.

**Case presentation:**

We present the case of a 30-year-old Caucasian woman who presented with unusually persistent bilateral areas of lung consolidation on serial radiographs. Pulmonary sarcoidosis was diagnosed on transbronchial lung biopsy five months after the initiation of treatment with interferon-β for multiple sclerosis.

**Conclusions:**

Sarcoidosis should be considered in the differential diagnosis of a patient who develops clinical or radiological pulmonary disease while undergoing interferon therapy. It is important to note that interferon-induced sarcoidosis, though usually seen in cases with interferon-α, can occur with interferon-β. Neurologists managing patients with multiple sclerosis should be aware of this association between interferon-β and sarcoidosis and promptly refer patients developing respiratory symptoms for further investigation.

## Introduction

Sarcoidosis is a chronic systemic disease characterized by noncaseating granuloma formation that commonly affects the lungs. In pulmonary sarcoidosis, typical changes on plain chest radiography include hilar lymphadenopathy or reticulonodular infiltrates or both.

The exact etiology of sarcoidosis remains unknown. The existence of an exaggerated immune response to unknown antigenic stimuli has been proposed [1]. A number of immune modulators such as interferon-γ have been implicated [2]. In recent years, with the increasing use of recombinant α and β interferon therapy for the treatment of various disorders, cases of interferon-associated sarcoidosis have been reported in the literature. The majority of these have been cases of interferon-α-induced sarcoidosis [3-5]. These are described in detail in the Discussion section.

We report the case of a 30-year-old Caucasian woman who presented with unusually persistent bilateral areas of lung consolidation on serial radiographs. Pulmonary sarcoidosis was diagnosed five months after the initiation of treatment with interferon-β for multiple sclerosis. To the best of our knowledge, this represents the first case of interferon-induced pulmonary sarcoidosis in a patient whose multiple sclerosis was treated with interferon-β.

## Case presentation

A 30-year-old Caucasian woman presented to hospital with a three-week history of influenza like symptoms, including joint and muscle aches, fatigue, fever, rigors and tachycardia. She denied breathlessness or productive cough. She had already received a course of antibiotics from her general practitioner. Her inflammatory markers were normal, but a chest radiograph showed bilateral patchy areas of consolidation. She was discharged with a further course of oral antibiotics for presumed pneumonia.

Her symptoms gradually resolved, but the radiological abnormalities persisted on serial chest radiographs. She was subsequently referred to our respiratory clinic six weeks after her initial presentation.

In the clinic, our patient reported feeling well, apart from dry cough and tiredness. Her medical history included childhood asthma, asymptomatic chronic idiopathic urticaria, irritable bowel syndrome and multiple sclerosis. She had been receiving treatment for multiple sclerosis with interferon-β for the previous five months at a dose of 44μg subcutaneously three times a week. Concern that the fever and influenza-like symptoms were possible side effects led to discontinuation of the interferon therapy, which was not resumed. She was also taking omeprazole, mebeverine and amitriptyline. Her family history was relevant for multiple sclerosis in her sister, hypothyroidism and polymyalgia rheumatica in her mother and type 2 diabetes mellitus in her father. She smoked 10 to 15 cigarettes a day. On examination, she had normal vital signs. There was no lymphadenopathy and she did not have any skin lesions. The results of cardiovascular examination were normal. Respiratory examination revealed an area of bronchial breathing on her left lung, but there was no wheeze or crepitations.

**Figure 1:**
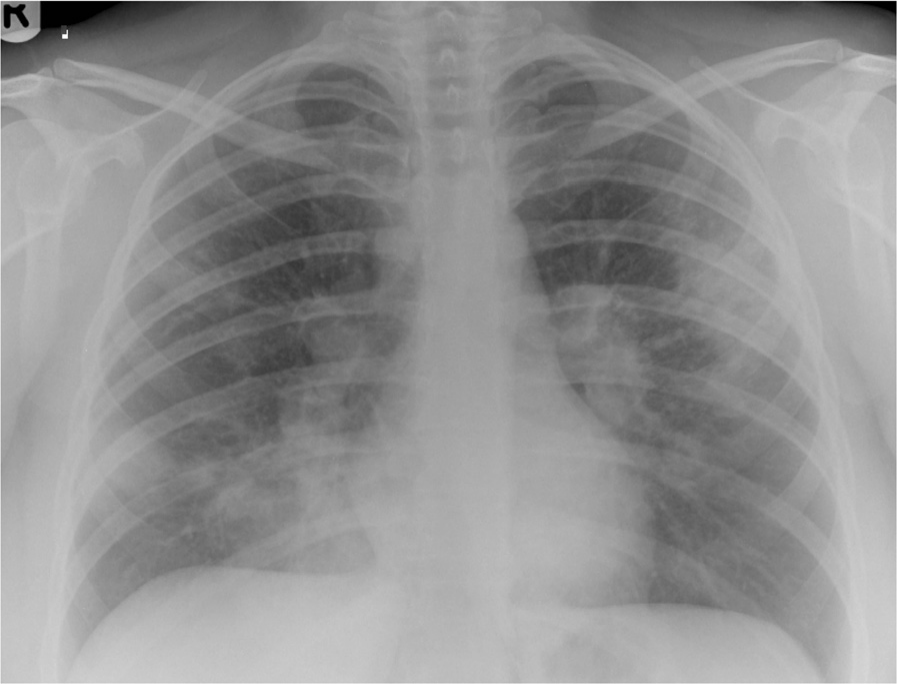
Plain chest radiograph demonstrating bilateral patchy air-space consolidation and prominent hilar shadows.

**Figure 2:**
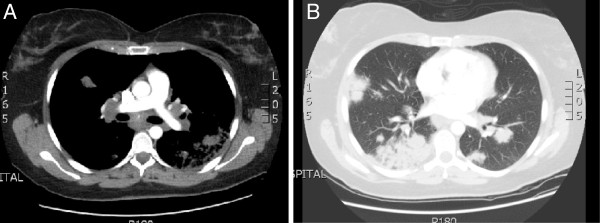
** Computed tomography scan of thorax. (a)** Extensive mediastinal and bilateral hilar lymphadenopathy. **(b)** Multifocal patchy air-space consolidation in both lungs.

At her initial presentation with presumed pneumonia, her full blood count, renal and liver function, and calcium and glucose levels were all normal. She had a C-reactive protein level of 11mg/L (normal range, less than 5mg/L). Normal results were found at the clinic for her thyroid function, an autoimmune screen - including levels of antinuclear antibodies, extractable nuclear antigen and antineutrophil cytoplasmic antibody - and her 24-hour urinary calcium levels. A plain chest radiograph (Figure 1) and a computed tomography scan of her thorax (Figure 2) are shown.

Pulmonary function testing revealed a forced expiratory volume in 1 second (FEV_1_) of 2.8L/sec (95% of predicted rate), a forced vital capacity (FVC) of 3.56 L (105% of predicted volume), an FEV_1_/FVC ratio of 94%, a transfer factor of the lung for carbon monoxide of 74% of the predicted capacity, and a carbon monoxide transfer coefficient of 85% of the predicted value.

**Figure 3:**
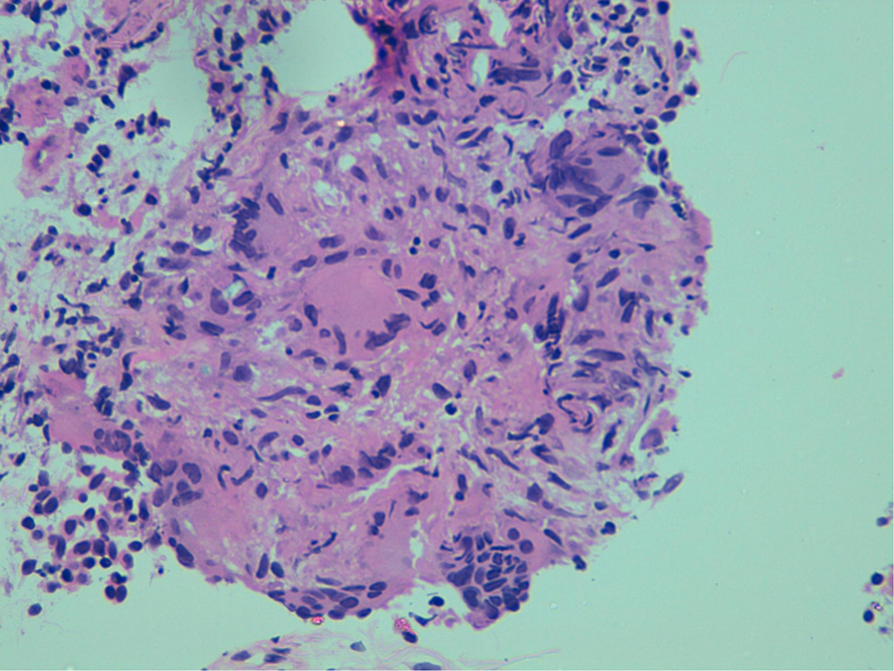
** Histology picture of a transbronchial lung biopsy specimen showing the presence of Langhans-type giant cells and noncaseating granulomas.** Hematoxylin and eosin stain; magnification ×40.

On bronchoscopy, the mucosa was fragile and hyperemic. Fluid from bronchoalveolar lavage was negative for malignancy and infection. Acid-fast bacilli were not detected on microscopy or culture of the bronchoalveolar lavage specimen. A transbronchial lung biopsy specimen showed the presence of histiocytes, giant cells and noncaseating granulomas (Figure 3), consistent with a diagnosis of pulmonary sarcoidosis.

Although our patient felt better after the interferon-β was discontinued, she continued to report fatigue and persistent dry cough. She had persistent and extensive bilateral parenchymal changes in her lungs and some degree of impairment in her lung function test, including reduced transfer factor of the lung for carbon monoxide. For these reasons, she was started on 40mg of prednisolone daily. Within four weeks of treatment, there was significant resolution of the radiological abnormalities, her cough disappeared, and she reported reduced fatigue. She continued on a reducing regime of steroid therapy over the following six months and remained well with no flare-up of sarcoidosis. Her disease course was mild and resembled that of reported interferon-induced sarcoidosis cases, as discussed in the following section.

## Discussion

Sarcoidosis is a chronic systemic disease characterized by noncaseating granuloma formation that can affect almost any organ system and that commonly affects the lungs. The exact etiology of sarcoidosis remains unknown. An exaggerated immune response to unknown antigenic stimuli is thought to exist. Proposed antigens include infectious agents, such as mycobacteria, environmental agents and autoantigens [1]. In sarcoidosis, the affected organs appear to have a predominance of type 1 T helper cells (cluster of differentiation 4 T cells) and macrophages, resulting in granuloma formation. A number of immune modulators, such as interleukin-2, interleukin-12 and interferon-γ, have been implicated [2]. Interferon-γ is released from lung T lymphocytes and alveolar macrophages and activates pulmonary macrophages, contributing to granuloma formation [6].

Interferon-γ belongs to the class II interferon family. It has a different structure and binds to a different receptor than the class I interferons (α and β), and its gene is located on a different chromosome [7]. Although interferon-γ has been implicated in the pathogenesis of sarcoidosis [6], there is little evidence implicating other types of interferons. However, there is evidence that exogenously administered interferon-α and interferon-β can activate macrophages *in vitro*[8].

Recombinant interferons (α and β) are used as immunomodulators in the treatment of various conditions, including viral infections such as hepatitis B and C, malignancies such as lymphoproliferative disorders, renal cell carcinoma and melanoma, and multiple sclerosis (interferon-β). Adverse reactions to interferon therapy include non-specific symptoms, such as cough, generalized malaise, fever and arthralgia, and develop in up to one third of patients in the first months of treatment [9]. In addition, various autoimmune processes have developed in patients treated with interferons [9]. The diverse immunomodulatory effects of exogenously administered interferons have been reported to upset the balance between self-tolerance and autoimmunity. With increasing use of interferon therapy in recent years, cases of interferon-associated sarcoidosis have been reported in the literature.

To the best of our knowledge, the first pathologically proven case of interferon-induced sarcoidosis was reported in 1987 in a woman whose advanced renal cell carcinoma was treated with interferon-β [10]. Since then, numerous published cases have suggested a relationship between interferon treatment and induction, recurrence or exacerbation of sarcoidosis. Most of the reported cases involve patients whose hepatitis C infection was treated with pegylated interferon-α (often in combination with ribavirin).

A case series in 1998 reported a 5% incidence of sarcoidosis in a cohort of 60 patients whose chronic hepatitis C infection was treated with interferon-α in a randomized control trial [3]. In 1999, Pietropaoli *et al*. [4] published the case of a 50-year-old woman who developed sarcoidosis while her chronic myelogenous leukemia was being treated with interferon-α. What was interesting in that case was that the authors demonstrated a dose–response effect and a temporal relationship of the interferon treatment with sarcoidosis activity, concluding that interferon-α most likely triggered the manifestations of sarcoidosis in that patient. In 2006, Goldberg *et al*. [5] reviewed the literature in English and found 60 cases of sarcoidosis development after the institution of recombinant interferon-α therapy. The majority of patients received interferon-α therapy for hepatitis C infection, but some were treated for hepatitis B, lymphoproliferative malignancies and other hematological conditions. The most commonly affected organs were the lungs and the skin. The mean onset of development of sarcoidosis was 11.4 months after initiation of interferon therapy (range of 1 to 60 months). Interferon therapy was discontinued in the majority of patients with interferon-induced sarcoidosis and, as a result, the sarcoidosis resolved within months. A small proportion of patients received systemic corticosteroid treatment and had rapid resolution of the sarcoidosis. Interferon-induced sarcoidosis generally exhibited a relatively mild disease course. Interestingly, a few patients in the literature responded to a reduction in the dose of interferon with resolution of the signs of disease.

It is important to note that interferon-induced sarcoidosis has been reported in numerous cases with interferon-α treatment but in only very few with interferon-β. In fact, in our literature search, we identified only two cases in which treatment with interferon-β induced sarcoidosis: a woman with advanced renal cell carcinoma in 1987 [10] and a patient with multiple myeloma in 2005 [11]. To the best of our knowledge, our case report represents the first case of interferon-induced pulmonary sarcoidosis in a patient whose multiple sclerosis was treated with interferon-β. It is important to note that our patient had no medical history or features of sarcoidosis prior to interferon therapy. Sarcoidosis was diagnosed five months after the initiation of interferon-β therapy.

Lastly, it is worth mentioning that bronchiolitis obliterans organizing pneumonia has also been reported after treatment with interferon-α and interferon-β [12], and pulmonary infiltrates and eosinophilia with interferon-α [13]. Indeed, the above differential diagnoses would be compatible with the clinical presentation of our patient, but the diagnosis of sarcoidosis was confirmed on transbronchial lung biopsy.

## Conclusions

Our experience with this case demonstrates that sarcoidosis should be considered in the differential diagnosis of a patient who develops pulmonary disease (clinical or radiological) while undergoing interferon therapy and that the diagnosis can usually be confirmed by transbronchial lung biopsy. A review of the literature demonstrated that interferon-induced sarcoidosis exhibits a mild disease course and most patients have complete resolution of the disease within a few months of discontinuing interferon therapy, without the need for immunosuppressants. Patients treated with systemic corticosteroids show rapid disease resolution.

It is important to note that, although interferon-induced sarcoidosis is usually seen with interferon-α therapy, our patient developed the condition with interferon-β. Neurologists managing patients with multiple sclerosis should be aware of the association between interferon-β and sarcoidosis and promptly refer patients developing respiratory symptoms for further investigation.

## Consent

Written informed consent was obtained from the patient for publication of this case report and accompanying images. A copy of the written consent is available for review by the Editor-in-Chief of this journal.

## Competing interests

The authors declare that they have no competing interests.

## Authors’ contributions

NP was directly involved in the diagnostic workup and overall care of the patient and drafted the manuscript. ECT was directly involved in the diagnostic workup and overall care of the patient, served as the lead clinician, selected the images presented, and obtained written consent from the patient. Both authors read and approved the final version of the manuscript.
